# Enhanced Wear Behaviour of Spark Plasma Sintered AlCoCrFeNiTi High-Entropy Alloy Composites

**DOI:** 10.3390/ma11112225

**Published:** 2018-11-08

**Authors:** Martin Löbel, Thomas Lindner, Thomas Lampke

**Affiliations:** Materials and Surface Engineering Group, Institute of Materials Science and Engineering, Chemnitz University of Technology, D-09107 Chemnitz, Germany; th.lindner@mb.tu-chemnitz.de (T.L.); thomas.lampke@mb.tu-chemnitz.de (T.L.)

**Keywords:** HEA, high-entropy alloy, compositionally complex alloy (CCA), compositionally complex alloy, SPS, spark plasma sintering, solid lubricant, phase composition, microstructure, wear behaviour

## Abstract

High hardness and good wear resistance have been revealed for the high-entropy alloy (HEA) system AlCoCrFeNiTi, confirming the potential for surface protection applications. Detailed studies to investigate the microstructure and phase formation have been carried out using different production routes. Powder metallurgical technologies allow for much higher flexibility in the customisation of materials compared to casting processes. Particularly, spark plasma sintering (SPS) enables the fast processing of the feedstock, the suppression of grain coarsening and the production of samples with a low porosity. Furthermore, solid lubricants can be incorporated for the improvement of wear resistance and the reduction of the coefficient of friction (COF). This study focuses on the production of AlCoCrFeNiTi composites comprising solid lubricants. Bulk materials with a MoS_2_ content of up to 15 wt % were produced. The wear resistance and COF were investigated in detail under sliding wear conditions in ball-on-disk tests at room temperature and elevated temperature. At least 10 wt % of MoS_2_ was required to improve the wear behaviour in both test conditions. Furthermore, the effects of the production route and the content of solid lubricant on microstructure formation and phase composition were investigated. Two major body-centred cubic (bcc) phases were detected in accordance with the feedstock. The formation of additional phases indicated the decomposition of MoS_2_.

## 1. Introduction

High-entropy alloys (HEAs) are gaining importance in current research. Regardless of their complex composition with at least five elements, the formation of simple cubic solid solutions can be achieved [1]. One of the first mentioned HEA systems with a single phase face-centred cubic (fcc) structure is the equimolar alloy CoCrFeMnNi [2]. However, further investigations of several alloy systems with a variation of alloy compositions revealed the formation of a multiphase microstructure, partially including intermetallic phases. For multiphase HEAs, the term compositionally complex alloys (CCAs) was introduced [3].

The remarkable properties of HEAs have been explained by four “core effects”: high entropy, sluggish diffusion, severe lattice distortion, and “cocktail effect” [4,5]. The high-entropy effect enables the formation of multi-element solid solutions due to the reduction of Gibbs free energy and hence thermodynamic stabilisation [6]. The sluggish diffusion effect refers to a reduced diffusion coefficient and a higher activation energy compared to conventional alloys. Consequently, phase transformations can be suppressed and high-temperature phases are stabilised, resulting in improved creep resistance and high-temperature properties [7]. A strong lattice distortion is caused by differences in the atomic size, the crystal structures and the different bonding energy of the alloying elements. Hence, the dislocation movement is impeded, causing a high hardness and strength [6]. The “cocktail effect” emphasises mutual interactions among all components leading to excess quantities of properties. As a consequence, resulting properties cannot be predicted by the rule of mixture [6].

One of the most investigated HEA systems is AlCoCrCuFeNi and the copper-free derivate AlCoCrFeNi [8,9]. Distinct improvement of mechanical properties can be achieved by alloying with titanium. Due to its large atomic radius, the addition of titanium causes solid–solution strengthening and hence increased strength and hardness. Additionally, the formation of brittle phases was observed for a high content of titanium [10,11,12]. The wear resistance of the alloy system AlCoCrFeNiTi has been investigated under various conditions revealing an increased wear resistance, also in comparison with the steels EN 1.3505 and EN 1.3343 [11,13]. Although improved high-temperature properties of AlCoCrFeNiTi are expected, the wear behaviour has not yet been investigated at increased temperature.

A further improvement of wear behaviour can be achieved by lubrication. Solid lubricants can be applied to reduce the coefficient of friction (COF). Powder metallurgical technologies enable the incorporation of solid lubricants by processing powder blends. The suitability of different powder production routes for the production of homogeneous and spherical HEA feedstock has been investigated, whereas inert gas atomisation showed the best results [14]. Spark plasma sintering (SPS) technology is appropriate for the fast production of dense material. In comparison to other sintering techniques, the current activated process enables high heating rates and a low sintering temperature. The processed material exhibits a low porosity and the absence of grain coarsening [15,16]. The suitability of the SPS processing route for HEAs has already been mentioned [17,18], whereas the approach of incorporating solid lubricants has only been investigated for a few HEA systems [19,20]. Beside graphite, MoS_2_ is one of the most commonly used solid lubricants, showing good lubricating performance below 400 °C [21,22,23].

This study focused on enhancing the wear behaviour of the equimolar AlCoCrFeNiTi HEA by the addition of MoS_2_. Materials with a different amount of the solid lubricant were produced. Wear testing was conducted under dry sliding conditions at room temperature and 150 °C. The bearing steel EN 1.3505 (100Cr6) was analysed as reference. Furthermore, the influence of the solid lubricant on microstructure and phase formation was investigated in detail.

## 2. Materials and Methods

For the production of equimolar AlCoCrFeNiTi feedstock material, an inert gas atomisation process was applied. Argon was used as the process gas for the reduction of impurities and reactions. The powder was separated by air classification, whereas the fraction below 20 µm was utilised for SPS processing.

The powder was characterised in detail. In a first step, laser diffraction analysis was conducted in a Cilas 930 device (Cilas, Orléans, France) for the determination of the particle size distribution. Subsequently, metallographic cross-sections were prepared by standard metallographic procedures for the investigation of powder morphology and phase formation. A scanning electron microscope (SEM) LEO 1455VP (Zeiss, Jena, Germany) equipped with a backscattered electron (BSE) detector (Zeiss, Jena, Germany) for the visualisation of material contrast was used. The acceleration voltage was 25 kV. The chemical composition was measured with an energy-dispersive X-ray spectroscopy (EDS) system. A calibrated EDS GENESIS system (EDAX, Mahwah, NJ, USA) was applied for semi-quantitative analysis. The phase formation was investigated by X-ray diffraction (XRD). A D8 Discover diffractometer (Bruker AXS, Billerica, MA, USA) operating with Co Kα radiation (U: 40 kV; I: 40 mA) was used to measure in a diffraction angle (2θ) range of 20° to 130°. The measurement was conducted with a step size of 0.01° and 2.2 s/step. Due to the use of a 1D Lynxeye XE detector (Bruker AXS, Billerica, MA, USA), this corresponded to 422 s/step. For phase identification, the powder diffraction file (PDF) database 2014 (International Centre for Diffraction Data) was applied.

Commercially available MoS_2_ solid lubricant OKS 110 (OKS Spezialschmierstoffe GmbH, Maisach, Germany) in powder shape with a particle size of d99 = 15 µm was used. Powder blends of AlCoCrFeNiTi powder with mass fractions of 2, 5, 8, 10, and 15 wt % MoS_2_ were produced and subsequently mixed in a tumbling mixer WAB Turbula (Willy A. Bachofen AG, Muttenz, Switzerland) for 10 min. The compaction of the samples was carried out in an SPS KCE FCT—HP D 25-SI (FCT Systeme GmbH, Frankenblick, Germany). Therefore, a graphite tool with an inner diameter of 40 mm was used to produce cylindrical samples with a thickness of 8 mm. Graphite foils were placed between powder and die walls. To avoid undesired chemical reactions, the whole process was conducted under argon atmosphere. The processing temperature of 1050 °C was held constant for 15 min at a uniaxial pressure of 50 MPa. Subsequently, the samples were cooled unregulated within the SPS device.

For the investigation of the SPS sample’s microstructure, cross-sections were prepared by standard metallographic procedures and polished with an active oxide polishing suspension OP-S (Struers, Willich, Germany) in the last step. The cross-sections were investigated in SEM and the chemical composition of the sample without MoS_2_ addition was measured by EDS. Phase analyses of all samples were conducted by XRD. The microhardness HV0.5 was measured with a Wilson Tukon 1102 device (Buhler, Uzwil, Switzerland). The average value and standard deviation were calculated from ten single indents.

The investigations of the wear behaviour under sliding wear conditions were carried out in ball-on-disk tests, using an SRV-Tribometer (Optimol Instruments Prüftechnik GmbH, Munich, Germany). Sintered Al_2_O_3_ was used as counter-body material due to its high hardness and wear resistance. The applied parameters are summarised in Table 1.

The bearing steel EN 1.3505 (100Cr6) was investigated as a reference. The resulting wear depths were measured by contact stylus instrument with a Hommel Etamic T8000 device (Jenoptik, Villingen-Schwenningen, Germany). Wear tracks were investigated by SEM (LEO 1455VP), using a BSE detector. The average coefficient of friction (COF) and the standard deviation were calculated for the period starting at a test time of 60 s until the end of the test.

## 3. Results and Discussion

### 3.1. Feedstock Characterisation

**Figure 1:**
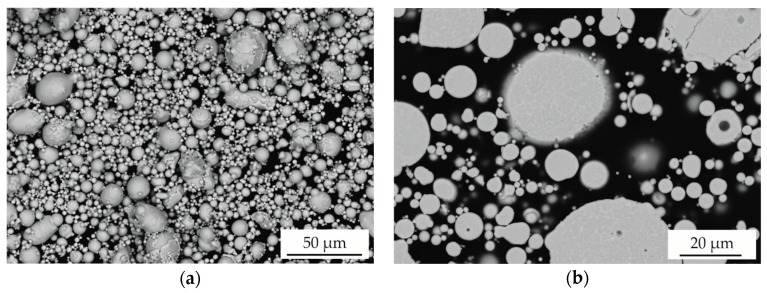
SEM (backscattered electron (BSE)) micrographs of (**a**) the surface and (**b**) the cross-section of atomised AlCoCrFeNiTi powder.

In a first step, the morphology and microstructure of the atomised AlCoCrFeNiTi powder was investigated in SEM. Images of the surface and cross-section of the powder are shown in Figure 1.

**Figure 2:**
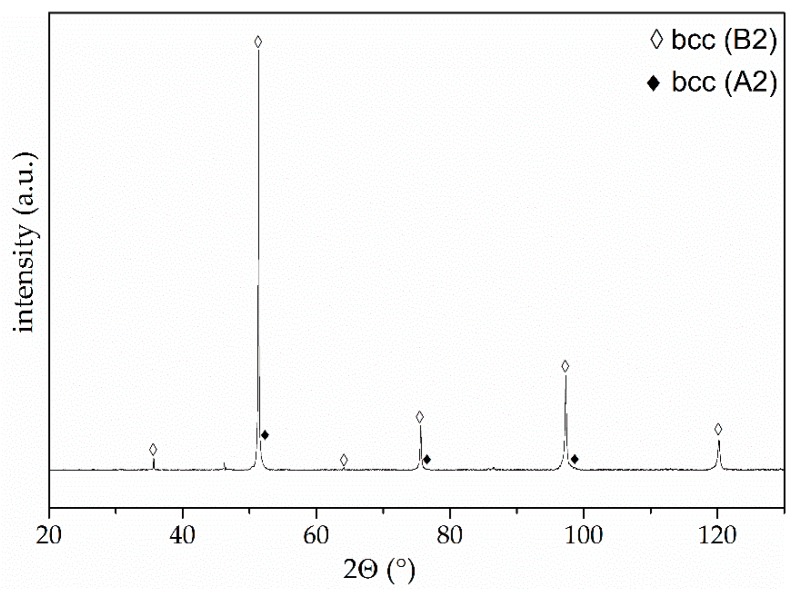
XRD diffractogram of inert-gas-atomised AlCoCrFeNiTi powder.

The particles have a predominantly spherical shape. A high content of fine particles with a diameter below 10 µm is revealed. In the cross-section, a contrast between different phase areas can be observed, indicating a deviating chemical composition due to the use of a BSE detector. The particle size distributions determined by laser diffraction analysis correspond to the SEM micrographs. A mean particle size (d50) of 9 µm and a particle size range (−d90 + d10) of −21 + 3 µm was determined. A phase analysis of the feedstock powder is shown in Figure 2.

The high-intensity diffraction peaks can be ascribed to a chemically ordered bcc phase with B2 structure. For the main diffraction peaks at 51.4° and 97.3°, a shoulder towards higher diffraction angles occurs, indicating the overlapping with the diffraction peaks of a chemically disordered bcc phase with A2 structure. A minor content of this phase is present in the investigated powder, which corresponds to the microstructural investigations, revealing the formation of a multiphase microstructure. Powder of the same alloy with a bigger particle size exhibits distinct diffraction peaks of the bcc (A2) phase (not shown here), indicating a higher phase content. For the fine powder fraction, which was cooled down faster in the atomisation process, suppression of the bcc (A2) occurs. Furthermore, the presence of the bcc (A2) phase was proven for arc-melted bulk samples. Another phase which was detected for coarser powder and bulk samples is the centred cluster (cc) with A12 structure type [10]. For the fine and fast-cooled powder, the presence of this phase cannot be distinctly proven.

### 3.2. Composite Characterisation

**Figure 3:**
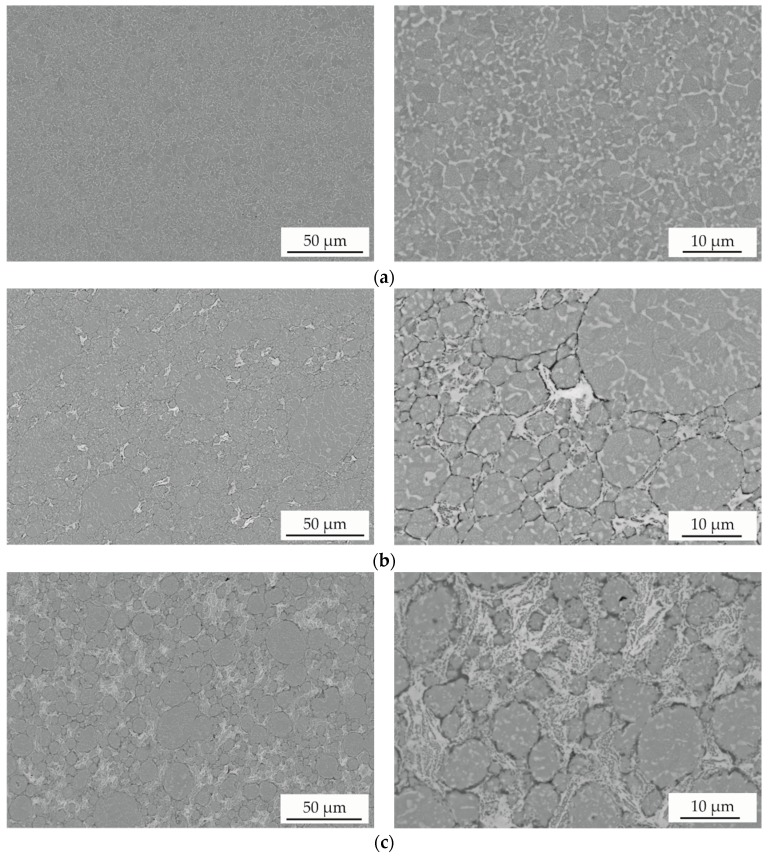
SEM images (BSE) of (**a**) AlCoCrFeNiTi and AlCoCrFeNiTi composites produced by spark plasma sintering (SPS) with (**b**) 5 wt % and (**c**) 15 wt % MoS_2_.

For the investigation of the composite’s microstructure, cross-sections were prepared and investigated by SEM. Images of the AlCoCrFeNiTi sample and of the composites with a MoS_2_ content of 5 and 15 wt % are shown in Figure 3. The AlCoCrFeNiTi sample without the addition of MoS_2_ exhibits a dense microstructure. At higher magnification, a multiphase microstructure can be observed in accordance with the atomised powder. Furthermore, boundaries of the feedstock powder can be recognized. For the composite with a low amount of MoS_2_ (5 wt %), an inhomogeneous distribution of the solid lubricant occurs. The boundaries between the HEA material and the solid lubricant can be visualised at a higher magnification. A multiphase microstructure is observed within the HEA material by a distinct BSE contrast. The composite with the maximum MoS_2_ content (15 wt %) shows a homogeneous distribution of the solid lubricant. Within the solid lubricant a distinct BSE contrast occurs, indicating a multiphase state and a decomposition of MoS_2_ during the SPS process.

The AlCoCrFeNiTi SPS sample (x = 0) exhibits a microhardness value of 706 ± 12 HV0.5. To evaluate possible variations of material composition during the SPS processing, the chemical composition of the atomised powder and the SPS sample was measured using EDS. The results are summarised in Table 2.

**Figure 4:**
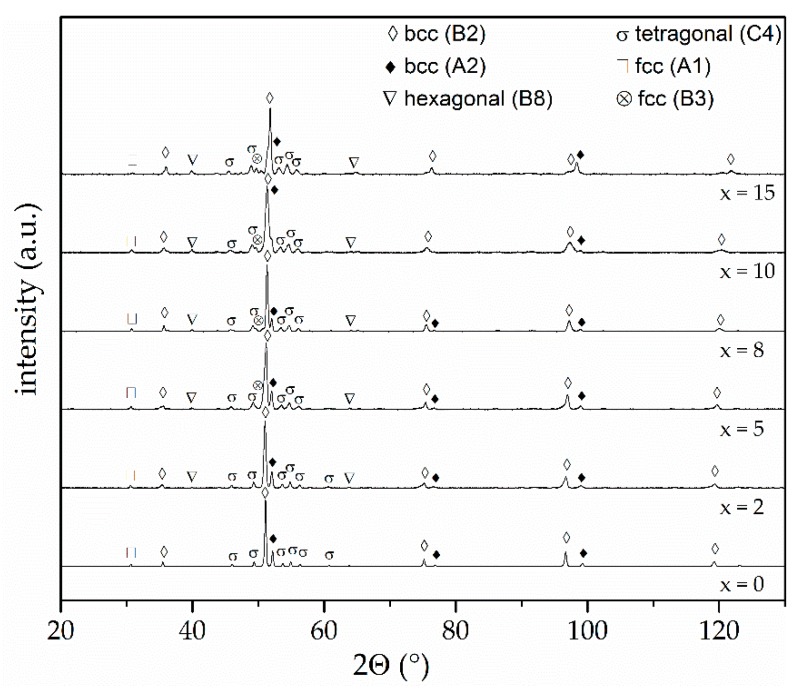
XRD diffractograms of AlCoCrFeNiTi composites produced by SPS, with varying MoS_2_ content (x in wt %).

The measured chemical composition of the atomised powder corresponds well to the nominal composition. A distinct deviation exceeding 1 at % occurs only for the aluminium content. After the SPS processing, no significant change of chemical composition can be observed in comparison to the atomised powder. The phase composition of the AlCoCrFeNiTi sample and the composites was investigated by XRD. Resulting diffractograms are depicted in Figure 4.

The XRD diffractogram of the AlCoCrFeNiTi SPS sample (x = 0) shows main diffraction peaks of the chemically ordered bcc (B2) phase, which are in accordance with the utilised feedstock. Furthermore, distinct peaks of the chemically disordered bcc (A2) phase appear. At a diffraction angle of 31°, an additional peak is found, which is characteristic for an fcc (A1) phase. Further diffraction peaks of this phase overlap with the bcc (B2) phase. Since the fcc (A1) phase is not detected for the feedstock, the suppression can be explained by the fast cooling within the atomisation process. Further peaks are found for the SPS state, which can be ascribed to a tetragonal σ-phase. The formation of a σ-phase has already been proven by Moravcik et al. for an AlCoCrFeNiTi_0.5_ alloy produced by SPS. Subsequent heat treatment of the SPS samples resulted in dissolution, showing that the formation sensitively depends on manufacturing conditions [24].

For the composite samples (x ≥ 2), the same major phases are formed (bcc (B2), bcc (A2), fcc (A1), σ). However, additional diffraction peaks occur, indicating the formation of further phases. Minor peaks at diffraction angles of 39.9° and 63.8° indicate the formation of an additional hexagonal (B8) phase. The main peak of this phase overlaps with the major bcc (B2) phase. Both FeS and the hexagonal (B8) phase exhibit the same lattice structure. However, the lattice parameters are significantly different, which might be caused by the diffusion of other elements into the lattice. With a further increase in MoS_2_ content (x ≥ 5), additional diffraction peaks occur. The peak at a diffraction angle of 49.7° can be ascribed to an fcc (B3) phase with a ZnS structure type. This phase only exhibits a few high-intensity diffraction peaks. The maximum at 45.5° overlaps with a diffraction peak of the σ-phase. The diffraction peak at 49.7° becomes more distinct with increased MoS_2_ content, indicating a higher phase content. No distinct diffraction peaks of MoS_2_ can be detected, showing that a decomposition of the solid lubricant occurs, which corresponds to the microstructural investigations. A summary of detected phases and major crystallographic information is presented in Table 3.

**Figure 5:**
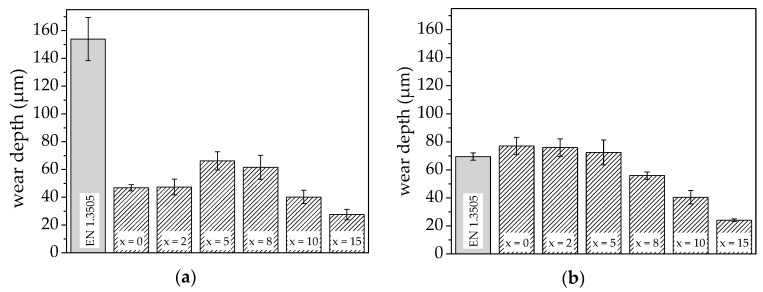
Wear depths of bearing steel EN 1.3505, AlCoCrFeNiTi sample, and composites produced by SPS, with a MoS_2_ content of x wt % in the ball-on-disk test at (**a**) room temperature and (**b**) 150 °C.

The wear behaviour of the bearing steel EN 1.3505, the AlCoCrFeNiTi sample, and the composites was investigated under sliding wear conditions at room temperature and 150 °C. The resulting wear depths are shown in Figure 5.

The investigation of sliding wear behaviour at room temperature reveals no influence of a minor amount of MoS_2_ (x ≤ 2) on the resulting wear depth of the AlCoCrFeNiTi samples. Adding more MoS_2_ causes a slight increase of the wear depth. However, a reduction of the wear depth can be achieved for a further increased MoS_2_ content (x ≥ 10), whereas the lowest wear depth and consequently highest wear resistance is achieved for the composite with a MoS_2_ content of 15 wt %. In comparison to the bearing steel (EN 1.3505), all investigated AlCoCrFeNiTi composites exhibit a distinctly lower wear depth and hence higher wear resistance.

For the AlCoCrFeNiTi sample (x = 0) tested at a temperature of 150 °C, the wear depth increases in comparison to the same sample tested at room temperature. With the addition of MoS_2_ up to a content of 5 wt %, no distinct variation of wear depth occurs. For further increased MoS_2_ contents, a decrease of the wear depth can be observed. The lowest value is measured for the composite with a MoS_2_ content of 15 wt %. The bearing steel (EN 1.3505) exhibits a clearly reduced wear depth in comparison to the investigations at room temperature, indicating the formation of a protective tribofilm. However, a reduction of the wear depth can be achieved for the HEA composites with a MoS_2_ content above 8 wt %.

**Figure 6:**
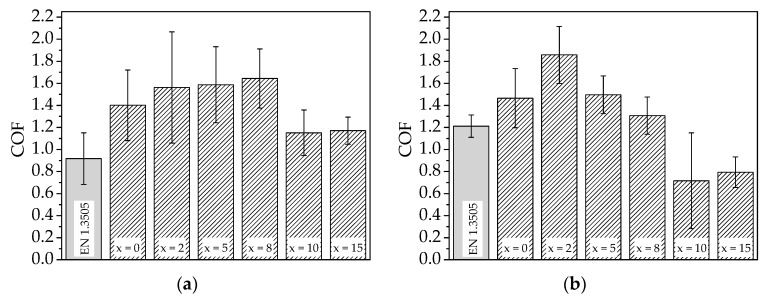
Coefficient of friction (COF) of bearing steel EN 1.3505, AlCoCrFeNiTi sample, and composites produced by SPS, with a MoS_2_ content of x wt % in the ball-on-disk test at (**a**) room temperature and (**b**) 150 °C.

Besides the increase of wear resistance, a decrease of COF is intended. The measured average COF and standard deviations are shown in Figure 6.

For the AlCoCrFeNiTi SPS sample (x = 0), an average COF of 1.40 was measured. With the addition of a minor amount of MoS_2_, no distinct change of COF occurs (x ≤ 8). These samples exhibit a high standard deviation, showing that no continuous lubrication is achieved. Especially for the composite with x = 2, a high standard deviation is measured. This behaviour corresponds to the microstructural investigations, revealing an inhomogeneous distribution of the solid lubricant for low contents. As a result, an interrupted lubrication occurs. With a further increase of MoS_2_ content to x = 10, the COF distinctly decreases. The reduced standard deviation indicates a more homogeneous lubrication. For the composite with the highest amount of MoS_2_ (x = 15), no further decrease of the COF can be observed. However, a further decrease of the standard deviation can be noticed. In comparison to the bearing steel EN 1.3505, all investigated samples exhibit a higher COF, under sliding wear conditions at room temperature.

In comparison to the wear tests at room temperature, the COF of the lubricant-free SPS sample (x = 0) does not significantly change with increased temperature (COF = 1.46). An increase of the COF is observed for x = 2. The further addition of MoS_2_ causes a reduction of the COF, whereas the COF is significantly reduced for x = 10. However, the high standard deviation of the COF indicates that fluctuating lubrication occurs. For the sample with the highest MoS_2_ content (x = 15), the standard deviation is reduced, indicating a continuous lubrication. In comparison to the bearing steel EN 1.3505 and the AlCoCrFeNiTi sample (x = 0), the composites with x = 10 and 15 exhibit a reduced COF under sliding wear conditions at 150 °C.

**Figure 7:**
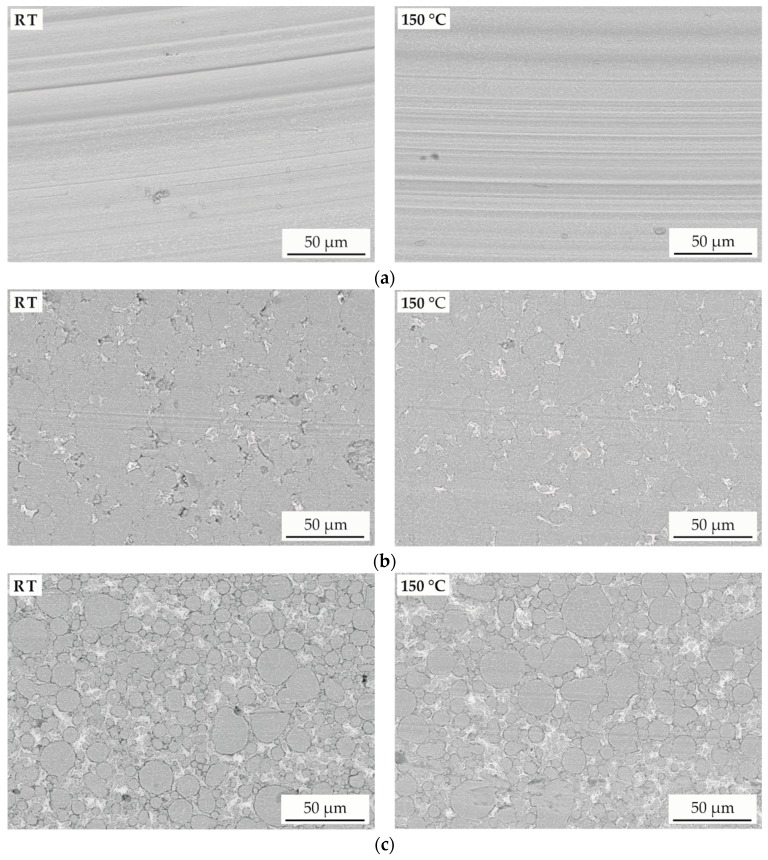
SEM images (BSE) of the AlCoCrFeNiTi composites produced by SPS with a MoS_2_ content of (**a**) 0 wt %, (**b**) 5 wt %, and (**c**) 15 wt % after the ball-on-disk test at room temperature (RT) and 150 °C.

For the investigation of the wear behaviour, the wear tracks were examined by SEM. Images of the surface after the ball-on-disk tests are shown in Figure 7. Deep grooves appear at the surface of the SPS samples without solid lubricant (x = 0) under both testing temperatures. Additionally, no spalling or brittle behaviour can be observed. Due to the application of a hard Al_2_O_3_ counterpart, predominantly abrasive wear occurs. Similar results independent of the temperature prove that there is no distinct change in the wear behaviour.

A change in wear mechanism can be observed for the SPS samples with solid lubricant. Smaller grooves appear and additional spalling can be determined for a low content of solid lubricant. For the sample x = 5 tested at room temperature, breakouts of the lubricant are visible. The absence of breakouts in the cross-sections (Figure 3) proves that spalling is caused by wear. A low content of lubricant and surface spallation result in an increased wear depth in comparison to the reference without MoS_2_ (Figure 5). Furthermore, a high standard deviation of the COF occurs, caused by an inhomogeneous lubrication. Spallation can be prevented by increasing the testing temperature to 150 °C. However, the underlying mechanisms are not yet fully understood. A possible explanation for the better integration of the solid lubricant is the differences in thermal expansion causing mechanical clamping. With the absence of spallation, enhanced wear resistance is observed. This can be associated with a gradual lubrication improvement for the increased MoS_2_ content. For both temperatures, the SPS sample x = 15 shows the lowest wear depth and a low COF. A homogeneous distribution of the solid lubricant between the primary particles can be observed after the wear investigation. The absence of spalling can be explained by the better interconnection of the lubrication phase. A decreasing standard deviation of the COF indicates a more homogeneous wear behaviour with good lubrication.

## 4. Summary and Conclusions

The equimolar alloy AlCoCrFeNiTi and composites comprising solid lubricants were successfully processed by SPS. Samples with dense microstructure and good bonding between different microstructural domains were produced. A multiphase microstructure with two major bcc phases occurred for the feedstock material and the SPS samples. However, two minor additional phases appeared after the SPS process. Composite samples of AlCoCrFeNiTi and the solid lubricant MoS_2_ were produced with the intention to increase the wear resistance and reduce the COF. A homogeneous distribution of the solid lubricant was achieved. Phase analyses of the composite samples revealed a decomposition of MoS_2_ by the formation of two additional phases for high contents of solid lubricant. Dry sliding wear behaviour was investigated in the ball-on-disk tests at different temperatures. An increase of the wear resistance and a decrease of the COF were achieved for the composites with a MoS_2_ content of at least 10 wt %. This amount was required for an efficient and homogeneous lubrication. For lower contents, a weakening of the composites occurred, resulting in a high wear depth and COF due to the inhomogeneous distribution of the solid lubricant. Furthermore, spalling occurred depending on the test conditions. In comparison to the bearing steel EN 1.3505, a higher wear resistance was observed at both test temperatures for the composites with a minimum MoS_2_ content of 10 wt %. The investigation of wear tracks revealed the presence of predominantly abrasive wear. Only minor spalling occurred for these composites, indicating a good bonding of the SPS samples.

The good wear properties of the HEA composites make them a promising material for wear protection applications and bearing materials. Further investigations must be conducted to investigate the wear behaviour at higher temperatures and for higher contents of solid lubricants.

## Figures and Tables

**Table 1:** Wear test parameters.

Ball-on-Disk Test	
Force	20 N
Radius	5 mm
Speed	96 rpm
Cycles	15,916
Counter Body	Al_2_O_3_
Diameter	6 mm
Temperature	22 °C; 150 °C

**Table 2:** Chemical composition of atomised AlCoCrFeNiTi powder and SPS sample in at %, measured by EDS.

Sample	Atomised Powder	SPS
Al	18.3	18.1
Co	16.0	16.3
Cr	16.9	16.8
Fe	16.7	16.5
Ni	16.1	16.4
Ti	16.0	16.0

**Table 3:** Phases detected by XRD for the atomised powder (P) and the AlCoCrFeNiTi composites produced by SPS with a MoS_2_ content of x in wt %.

Struktur-bericht	Lattice	Structure Type	Pearson Symbol	Space Group	Lattice Parameter (Å)	Detected for x
B2	bcc	CsCl	cP2	$\mathrm{Pm}\bar{3}$m (221)	2.92	P, 0, 2, 5, 8, 10, 15
A2	bcc	W	cI2	Im3¯m (229)	2.88	P, 0, 2, 5, 8, 10, 15
A1	fcc	Cu	cF4	$\mathrm{Fm}\bar{3}$m (225)	5.86	P, 0, 2, 5, 8, 10, 15
C4	tetragonal	Ti0_2_	tP6	P4_2_/mnm (136)	a = 8.86 c = 4.60	P, 0, 2, 5, 8, 10, 15
B8	hexagonal	NiAs	hP4	P63/mmc (194)	a = 3.39 c = 5.93	2, 5, 8, 10, 15
B3	fcc	ZnS	cF8	$F\bar{4}$3m (216)	-	5, 8, 10, 15
